# Comparative genotypic and pathogenic examination of *Campylobacter concisus *isolates from diarrheic and non-diarrheic humans

**DOI:** 10.1186/1471-2180-11-53

**Published:** 2011-03-15

**Authors:** Lisa D Kalischuk, G Douglas Inglis

**Affiliations:** 1 Agriculture and Agri-Food Canada, 5403-1st Avenue South, Lethbridge, AB T1J 4B1, Canada

## Abstract

**Background:**

*Campylobacter concisus *is an emerging enteric pathogen, yet it is commonly isolated from feces and the oral cavities of healthy individuals. This genetically complex species is comprised of several distinct genomospecies which may vary in pathogenic potential.

**Results:**

We compared pathogenic and genotypic properties of *C. concisus *fecal isolates from diarrheic and healthy humans residing in the same geographic region. Analysis of amplified fragment length polymorphism (AFLP) profiles delineated two main clusters. Isolates assigned to AFLP cluster 1 belonged to genomospecies A (based on genomospecies-specific differences in the 23S rRNA gene) and were predominantly isolated from healthy individuals. This cluster also contained a reference oral strain. Isolates assigned to this cluster induced greater expression of epithelial IL-8 mRNA and more frequently contained genes coding for the zonnula occludins toxin and the S-layer RTX. Furthermore, isolates from healthy individuals induced greater apoptotic DNA fragmentation and increased metabolic activity than those from diarrheic individuals, and isolates assigned to genomospecies A (of which the majority were from healthy individuals) exhibited higher haemolytic activity compared to genomospecies B isolates. In contrast, AFLP cluster 2 was predominated by isolates belonging to genomospecies B and those from diarrheic individuals. Isolates from this cluster displayed greater mean epithelial invasion and translocation than cluster 1 isolates.

**Conclusion:**

Two main genetically distinct clusters (i.e., genomospecies) were identified among *C. concisus *fecal isolates from healthy and diarrheic individuals. Strains within these clusters differed with respect to clinical presentation and pathogenic properties, supporting the hypothesis that pathogenic potential varies between genomospecies. ALFP cluster 2 isolates were predominantly from diarrheic patients, and exhibited higher levels of epithelial invasion and translocation, consistent with known roles for these factors in diarrhoeal disease. Conversely, isolates from healthy humans and AFLP cluster 1 or genomospecies A (which were predominantly isolated from healthy humans) exhibited increased haemolytic ability, apoptotic DNA fragmentation, IL-8 induction, and/or carriage of toxin genes. Given that this cluster contains an oral reference strain, it is possible that some of the AFLP cluster 1 isolates are periodontal pathogens and may cause disease, albeit via a different mechanism than those from AFLP cluster 2.

## Background

*Campylobacter *species are one of the most common causes of human enteritis in North America (Centers for Disease Control and Prevention, U.S. Department of Agriculture, and Food and Drug Administration Collaborating Sites Foodborne Disease Active Survey Network [FoodNet]; Public Health Agency of Canada website, http://dsol-smed.phac-aspc.gc.ca/dsol-smed/ndis/diseases/camp_e.html). While *Campylobacter jejuni *and *Campylobacter coli *are the most commonly isolated species, studies have also implicated 'cryptic' species within the genus, such as *Campylobacter concisus*, as causal agents of acute enteritis [1-4]. Compared to *C. jejuni*, *C. concisus *is fastidious to isolate as it is often sensitive to selective antimicrobial agents commonly-used in conventional isolation media, and generally requires a hydrogen-enriched atmosphere and a prolonged incubation period for growth [5]. As such, it is rarely cultured by standard isolation methods employed by many diagnostic facilities. Although knowledge of its clinical importance is limited, *C. concisus *has been cited as an emerging human pathogen [5,6].

*Campylobacter concisus *was originally isolated from periodontal lesions [7]. However, its pathogenic role in oral cavity infections remains uncertain, since it can also be isolated from healthy gingiva [8]. Additionally, *C. concisus *has been isolated from the feces of diarrheic patients [1-4], often in the absence of known pathogens. However, the bacterium is also frequently isolated from feces of asymptomatic patients, which has lead to the conclusion that it may be part of the normal intestinal microbiota [9,10]. Some evidence indicates that *C. concisus *may be an opportunistic pathogen. For example, Engberg et al. [9] observed that *C. concisus *was predominantly isolated from pediatric, elderly, and immunocompromised patients, in contrast to *C. jejuni *and *C. coli *which are typically isolated from diarrheic patients of all ages. Consequently because of its association with diarrheic, healthy, and immunocompromised patients, the specific role of *C. concisus *as a primary intestinal pathogen has yet to be firmly established.

*Campylobacter concisus *is a heterogeneous species complex comprised of several phenotypically indistinguishable but genetically distinct taxa ("genomospecies"). Numerous methods can be used to genetically separate the genomospecies, including PCR analysis of the 23S rRNA gene [11] and cluster analysis of amplified fragment length polymorphism (AFLP) or random amplified polymorphic DNA (RAPD) profiles [1,2]. Based on these typing methods, at least two main *C. concisus *genomospecies have been identified [1,2,4,11].

Differences in pathogenicity amongst distinct genomospecies of some bacterial taxa [12,13] support the notion that certain *C. concisus *genomospecies may be more likely than others to cause intestinal disease. While an early study by Van Etterijck et al. [10] concluded that *C. concisus *was not pathogenic given similar isolation rates from diarrheic and healthy children, genetic diversity of the isolates with respect to clinical presentation was not considered. A more recent study showed that isolates from healthy individuals were genetically distinct from those of diarrheal origin; however, differences in epithelial cytotoxicity between the two groups were not evident [2]. Additionally, cluster analysis of diarrheic isolate AFLP profiles delineated two main *C. concisus *genomospecies (designated genomospecies 1 and 2), which where characterized by type strains of oral and diarrheal origin, respectively [1]. Genomospecies 2 isolates were more frequently isolated from the stool of patients presenting with diarrhea in which no other pathogens were found, and bloody diarrhea was associated only with genomospecies 2 isolates. While these studies suggest that distinct *C. concisus *genomospecies may vary in their pathogenic ability, this has yet to be empirically examined.

Our understanding of *Campylobacter *pathogenesis is based primarily on *C. jejuni*. Its small, spiral shape coupled with flagella-mediated motility, allow *C. jejuni *to penetrate intestinal mucus [14] where it can then adhere to and invade intestinal epithelial cells. This bacterium can also translocate across the intestinal epithelium via a paracellular mechanism involving disruption of epithelial tight junctions [15,16] or via a lipid raft-mediated transcellular mechanism [17]. *C. jejuni *also causes cellular cytotoxicity through the production of various toxins; cytolethal distending toxin (CDT) is a well-characterized toxin produced by most strains. The cytolethal distending toxin blocks cell proliferation in the G2/M phase resulting in cellular distension leading to the induction of apoptotic cell dealth [18]. This bacterium also induces intestinal epithelial secretion of interleukin-8 (IL-8), a pro-inflammatory chemoattractant that recruits neutrophils to the site of infection [19]. Cytolethal distending toxin-like activity has been reported for a majority of clinical *C. concisus *isolates [2], however the role that this toxin plays in pathogenesis is unknown. A membrane-bound haemolytic phospholipase is also produced by most clinical *C. concisus *isolates [20]. In addition, *C. concisus *genes coding for zonnula occludins toxin (*zot*) and a surface-layer protein belonging to the RTX (repeats in the structural toxins) family (*S-layer RTX*) have been recently identified [21]. Zonnula occludins toxin was first recognized as a toxin of *Vibrio cholera*, and disrupts the integrity of the intestinal epithelial barrier by targeting tight junctions [22]. S-layer RTX is a pore-forming toxin that is also found in *Campylobacter rectus *[23], and toxins within this family are recognized as important virulence factors [24].

The present study examines the hypothesis that the two main *C. concisus *genomospecies exhibit differences in pathogenicity. To address this hypothesis, we compared genotypic and pathogenic properties of *C. concisus *fecal isolates from diarrheic and asymptomatic ("healthy") humans. Specifically, genotypes of isolates were compared by AFLP analysis and a genomospecies-specific 23S rRNA gene PCR assay. Numerous pathogenic properties were also assessed including: (i) intestinal epithelial adherence, invasion, and translocation; (ii) ability to disrupt epithelial permeability, cause apoptotic DNA fragmentation, affect metabolic activity, and induce IL-8; hemolytic and cytotoxic activities; and (iii) carriage of toxin genes encoding CDT, ZOT, and S-layer RTX proteins.

## Results

### Genotypes

Sequence analysis to confirm the identities of the clinical isolates indicated >99% 16S rRNA gene sequence similarity (near full-length) between the type strain *C. concisus *LMG7788 and all of the clinical isolates (GenBank accession numbers are listed in Table 1). Based on the genomospecies-specific PCR assay of the 23S rRNA gene [11], six and 12 of the 22 clinical *C. concisus *isolates were assigned to genomospecies A and B, respectively (Table 1). Three isolates generated PCR products for both genomospecies A and B primer sets (designated "A/B"), and one isolate did not amplify with either primer set (designated "X"). The type strain, LMG7788, was assigned to genomospecies A, consistent with previous observations [2]. *Campylobacter concisus*-specific PCR of the *cpn*60 gene was strongly positive for 21 isolates including the type strain and weakly positive for two isolates. Weak PCR products were likely due to mismatching of the PCR primers with their target gene (due to DNA sequence divergence), resulting in inefficient PCR amplification.

**Table 1:** *Campylobacter concisus *isolates.

Isolate	Source	Genomospecies^a^	*cpn*60^b^	GenBank^c ^Accession #
CHRB6	Feces, diarrheic human	B	+	HM_536958.0
CHRB39	Feces, diarrheic human	A/B	+	n/a
CHRB318	Feces, diarrheic human	B	+	HM_536953.0
CHRB563	Feces, diarrheic human	A/B	+	HM_536957.0
CHRB1462	Feces, diarrheic human	B	+	HM_536942.0
CHRB1569	Feces, diarrheic human	B	+	HM_536943.0
CHRB1609	Feces, diarrheic human	A	+	HM_536944.0
CHRB1656	Feces, diarrheic human	B	+	HM_536945.0
CHRB1794	Feces, diarrheic human	A/B	+	HM_536946.0
CHRB2004	Feces, healthy human	A	+	HM_536947.0
CHRB2011	Feces, healthy human	A	+	HM_536948.0
CHRB2050	Feces, diarrheic human	A	+	HM_536949.0
CHRB2167	Feces, diarrheic human	B	+	n/a
CHRB2370	Feces, diarrheic human	B	+	HM_536950.0
CHRB2691	Feces, diarrheic human	B	+	HM_536951.0
CHRB2880	Feces, diarrheic human	B	+	n/a
CHRB3152	Feces, diarrheic human	B	W	HM_536952.0
CHRB3235	Feces, healthy human	X	W	HM_536954.0
CHRB3287	Feces, healthy human	A	+	HM_536955.0
CHRB3290	Feces, healthy human	A	+	HM_536956.0
CHRB3559	Feces, diarrheic human	B	+	n/a
CHRB3612	Feces, diarrheic human	B	+	n/a
				
LMG7788	Type strain, gingival sulcus	A	+	DQ_174166.1

^a ^Genomospecies determined using PCR assay for *C. concisus *23S rRNA gene. A/B indicates amplification with primer sets for both genomotype A and B. × indicates lack of PCR amplification with either primer set.

^b ^+ indicates PCR amplification of *cpn*60 gene; w indicates weak PCR amplification.

^c ^Near full-length 16S rRNA gene sequence.

**Figure 1:**
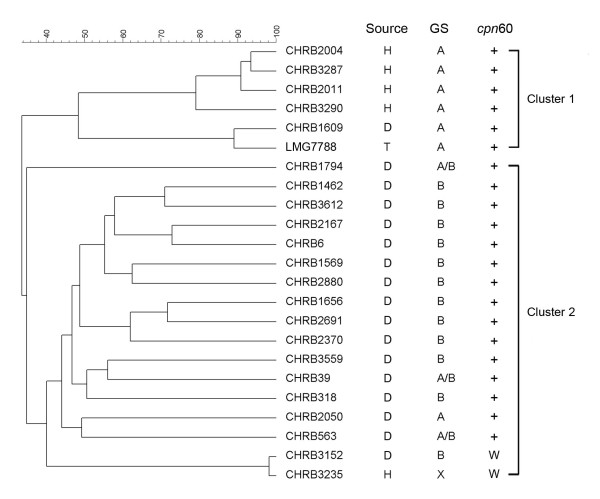
**Dendrogram of AFLP profiles derived using the unweighted-pair group average linkage of Pearson-product-moment correlation coefficients from 22 *Campylobacter concisus *fecal isolates (designated CHRB) and the type strain (LMG7788)**. The bar indicates percentage similarity. LMG, Culture Collection of the Laboratorium voor Microbiologie, Gent, Belgium. H, healthy humans. D, diarrheic humans. T, type strain. GS, genomospecies as determined by PCR assay of the 23S rRNA gene (2). A, genomospecies A. B, genomospecies B. A/B, indicates positive PCR for both genomospecies A and B. X, indicates negative PCR for both genomospecies A and B. *cpn*, *C. concisus*-specific *cpn*60 PCR. +, positive PCR. W, weak positive PCR. -, negative PCR.

AFLP analysis indicated considerable genetic variability existed among the *C. concisus *isolates (Figure 1). Reproducibility between duplicate independent analyses of each isolate was 93.1 ± 3.6% (mean ± SD; Additional file 1). The isolates clustered into two phylotypes distinguished from each other at the 34% similarity level. All isolates assigned to AFLP cluster 1 belonged to genomospecies A and included the type strain plus five isolates that were obtained from healthy (n = 4) and diarrheic (n = 1) humans. Of the seventeen isolates assigned to AFLP cluster 2, 94% (16/17) were isolated from diarrheic stools, and 71% belonged to genomospecies B (n = 12) while 17% belonged to genomospecies A/B (n = 3), 6% belonged to genomospecies A (n = 1), and one isolate was unassigned.

### Adherence, invasion, and translocation

All *C. concisus *isolates exhibited comparable epithelial adherence to that of *C. jejuni *81-176 (Table 2). The mean adherence of isolates belonging to genomospecies A did not differ from that of isolates belonging to genomospecies B (6.00 ± 0.08 log_10 _CFU/ml, n = 6 versus 6.28 ± 0.20 log_10 _CFU/ml, n = 5, respectively; *P *= 0.20). Similarly, mean adherence did not differ between isolates from diarrheic individuals and isolates from healthy volunteers (6.25 ± 0.11 log_10 _CFU/ml, n = 9 versus 6.08 ± 0.14 log_10 _CFU/ml, n = 5, respectively; *P *= 0.35), nor was it different for isolates assigned to AFLP cluster 1 versus cluster 2 (5.00 ± 0.09 log_10 _CFU/ml, n = 5 versus 6.30 ± 0.11 log_10 _CFU/ml, n = 9, respectively; *P *= 0.09).

**Table 2:** Intestinal epithelial adherence, invasion, and translocation of *Campylobacter concisus *isolates^a^.

Isolate	AFLP cluster	Adherence (log_10 _CFU/ml)	Invasion (log_10 _CFU/ml)	Translocation (log_10 _CFU/ml)
CHRB2004	1	6.12 ± 0.30^b^	4.50 ± 0.19	4.31 ± 0.65^b^
CHRB3287	1	6.03 ± 0.28^b^	4.72 ± 0.11^b^	3.74 ± 0.18^b^
CHRB2011	1	6.11 ± 0.21^b^	4.62 ± 0.18	3.87 ± 0.31^b^
CHRB3290	1	5.63 ± 0.31^b^	3.09 ± 0.10	3.84 ± 0.22^b^
CHRB1609	1	6.06 ± 0.06^b^	4.44 ± 0.12	4.19 ± 0.40^b^
				
CHRB1794	2	6.30 ± 0.26^b^	4.53 ± 0.13	5.07 ± 0.82^b^
CHRB6	2	6.03 ± 0.03^b^	5.06 ± 0.22^b^	4.38 ± 0.96^b^
CHRB1569	2	5.82 ± 0.14^b^	4.60 ± 0.23	3.71 ± 0.16^b^
CHRB2691	2	6.13 ± 0.24^b^	4.55 ± 0.21	4.86 ± 0.63^b^
CHRB2370	2	6.43 ± 0.20^b^	5.25 ± 0.13^b^	4.74 ± 0.45^b^
CHRB2050	2	6.06 ± 0.06^b^	4.64 ± 0.11^b^	3.97 ± 0.44^b^
CHRB563	2	6.48 ± 0.39^b^	5.01 ± 0.18^b^	4.77 ± 0.45^b^
CHRB3152	2	6.97 ± 0.03^b^	5.86 ± 0.34^b^	4.64 ± 0.54^b^
CHRB3235	2	6.48 ± 0.26^b^	5.65 ± 0.40^b^	5.07 ± 0.28^b^
				
LMG7788	1	5.16 ± 0.29^b^	3.26 ± 0.19	4.00 ± 0.31^b^
*C. jejuni *81-176	--	6.26 ± 0.34	5.70 ± 0.12	5.41 ± 0.49

^a ^Data are means ± SEM, n = 3

^b ^Not significantly different from *C. jejuni *81-176 (*P *> 0.05)

Epithelial invasion for seven *C. concisus *isolates was equivalent to that of *C. jejuni *81-176, including one of five isolates from AFLP cluster 1 and six of nine isolates for AFLP cluster 2 (Table 2). Isolates from AFLP cluster 2 were more invasive than cluster 1 isolates (5.02 ± 0.16 log_10 _CFU/ml versus 4.27 ± 0.30 log_10 _CFU/ml, respectively; *P *= 0.03). Mean invasion did not differ between isolates from diarrheic and healthy humans (4.88 ± 0.15 log_10 _CFU/ml versus 4.52 ± 0.41 log_10 _CFU/ml, respecively; *P *= 0.33) or isolates belonging to genomospecies A and B (4.34 ± 0.25 log_10 _CFU/ml versus 5.06 ± 0.24 log_10 _CFU/ml, respectively; *P *= 0.07). Adherence and invasion were positively correlated (R^2 ^= 0.71; *P *< 0.001).

Epithelial translocation was not different for any of the *C. concisus *isolates relative to *C. jejuni *81-176 (Table 2). The mean translocation of *C. concisus *genomospecies B isolates was greater than isolates belonging to genomospecies A (4.46 ± 0.20 log_10 _CFU/ml versus 3.99 ± 0.09 log_10 _CFU/ml, respectively; *P *= 0.048), and isolates assigned to AFLP cluster 2 relative to cluster 1 (4.58 ± 0.16 log_10 _CFU/ml versus 3.99 ± 0.11 log_10 _CFU/ml, respectively; *P *= 0.03). Mean translocation between isolates from diarrheic and healthy humans did not differ (4.48 ± 0.15 log_10 _CFU/ml versus 4.17 ± 0.25 log_10 _CFU/ml, respecively; *P *= 0.26). Translocation and invasion were weakly correlated (R^2 ^= 0.27; *P *= 0.059). There were no differences in the initial or final TER between *Campylobacter*-treated monolayers and the sterile broth control, and the final TER of all monolayers remained greater than 1000 Ω × cm^2 ^(Additional file 2) indicating that translocation occurred across an intact epithelial barrier. Also, FITC-dextran permeability did not differ for monolayers treated with *C. concisus *relative to the sterile broth control (Additional file 2).

### Hemolysis, DNA fragmentation, cytotoxicity, and metabolic activity

All *Campylobacter *isolates exhibited hemolytic activity as defined by the percent lysis of sheep red blood cells compared to the positive control (= 100%; Table 3). Mean hemolysis for genomospecies A isolates was greater than for isolates belonging to genomospecies B (64.0 ± 4.9% and 45.2 ± 5.1%, respectively; *P *= 0.027). Mean hemolysis did not differ between isolates from healthy and diarrheic individuals (55.9 ± 8.2% versus 52.0 ± 5.2%, respectively; *P *= 0.68), nor between isolates assigned to AFLP clusters 1 and 2 (63.9 ± 6.0% versus 47.5 ± 5.0%, respectively; *P *= 0.06). There was an inverse correlation between hemolysis and invasion (R^2 ^= 0.74; *P *< 0.0001) and between hemolysis and adherence (R^2 ^= 0.43; *P *< 0.011). None of the *C. concisus *isolates caused significant epithelial cytotoxicity, whereas *Campylobacter jejuni *81-176 and H_2_O_2 _induced cytotoxicity in agreement with previous observations [25] (Table 3).

**Table 3:** Hemolysis, DNA fragmentation, cytotoxicity, and metabolic activity of *Campylobacter concisus *isolates^a^.

Isolate	AFLP Cluster	Hemolysis^b^ (%)	DNA fragmentation^c^ (A_370 _nm)	Cytotoxicity^c^ (%)	Metabolic activity ^c^ (% control)
CHRB2004	1	60.2 ± 14.4	1.84 ± 0.17^d^	1.23 ± 0.21	139.4 ± 7.4
CHRB3287	1	45.6 ± 16.9	1.83 ± 0.13^d^	1.48 ± 0.16	146.8 ± 9.2
CHRB2011	1	60.5 ± 9.8	1.63 ±.0.05^d^	0.88 ± 0.22	151.9 ± 7.5
CHRB3290	1	81.1 ± 4.5	1.91 ± 0.14^d^	0.94 ± 0.19	155.7 ± 2.3
CHRB1609	1	72.3 ± 9.4	1.37 ± 0.18	1.11 ± 0.34	144.5 ± 4.4
					
CHRB1794	2	70.9 ± 10.1	1.32 ± 0.19	1.42 ± 0.15	137.9 ± 2.9
CHRB6	2	41.2 ± 11.6	1.12 ± 0.26	1.43 ± 0.18	105.1 ± 26.2^e^
CHRB1569	2	47.0 ± 12.0	1.38 ± 0.17	1.29 ± 0.26	139.2 ± 7.0
CHRB2691	2	62.1 ± 14.3	1.62 ± 0.07^d^	1.89 ± 0.15	133.5 ± 10.3
CHRB2370	2	44.9 ± 12.0	1.69 ± 0.14^d^	1.46 ± 0.08	142.8 ± 6.5
CHRB2050	2	64.3 ± 15.4	1.41 ± 0.07	0.97 ± 0.15	131.0 ± 7.1
CHRB563	2	34.6 ± 13.9	1.55 ± 0.23^d^	1.25 ± 0.20	138.0 ± 10.2
CHRB3152	2	30.7 ± 15.4	1.89 ± 0.16^d^	1.28 ± 0.15	141.0 ± 6.0
CHRB3235	2	32.1 ± 18.6	1.69 ± 0.12^d^	1.14 ± 0.16	143.2 ± 6.3
					
LMG7788	1	61.5 ± 10.8	1.54 ± 0.08^d^	0.71 ± 0.10	140.8 ± 5.2
					
*C. jejuni *81-176	--	75.6 ± 3.7	1.68 ± 0.25^d^	4.53 ± 0.31^d^	143.7 ± 5.7
Broth control	--	0.44 ± 0.14	0.69 ± 0.12	0.96 ± 0.34	100
H_2_O_2_	--	--	1.38 ± 0.22	6.15 ± 1.66^d^	259.5 ± 13.5
Camptothecin	--	--	2.23 ± 0.40^d^	1.39 ± 0.28	177.5 ± 9.2

^a ^Data are means ± SEM, n = 3.

^b ^Percent total hemolysis of sheep erythrocytes for 1/8 dilution of *Campylobacter *inoculum.

^c ^Assays conducted using T84 monolayers

^d ^*P *< 0.05 relative to the broth control treatment.

^e ^Sloughing of epithelial cells noted in two of three repetitions.

Epithelial DNA fragmentation was significantly induced by all five *C. concisus *isolates from healthy individuals, 44.4% of the isolates from diarrheic humans (i.e., four of nine isolates), *C. concisus *LMG7788, *C. jejuni *81-176, and the apoptosis-inducing agent, camptothecin (Table 3). Greater mean DNA fragmentation was observed for isolates from healthy volunteers compared to diarrheic individuals (1.78 ± 0.05 A_370 _nm versus 1.48 ± 0.08 A_370 _nm, respectively; *P *= 0.021). There was no difference in DNA fragmentation between isolates belonging to genomospecies A and B (1.66 ± 0.10 A_370 _nm versus 1.54 ± 0.13 A_370 _nm, respecively; *P *= 0.45), nor between isolates in AFLP groups 1 and 2 (1.72 ± 0.10 versus 1.52 ± 0.08 A_370 _nm, respectively; *P *= 0.15).

Epithelial cells inoculated with isolates from AFLP cluster 1 exhibited higher metabolic activity (i.e., MTT value) than those inoculated with AFLP cluster 2 isolates (147.7 ± 2.8 versus 134.6 ± 4.0%, respectively; *P *= 0.04). Likewise, metabolic activity in epithelial cells inoculated with isolates from healthy individuals was higher than that for isolates from diarrheic individuals (147.4 ± 2.9% versus 134.7 ± 4.0%, respectively; *P *= 0.049). Mean metabolic activity did not differ between isolates from genomospecies A and B (144.9 ± 3.6% versus 132.3 ± 7.0%, respectively; *P *= 0.13). Metabolic activity was positively correlated with DNA fragmentation (R^2 ^= 0.47; *P *= 0.007).

### Expression of IL-8

All *C. concisus *isolates and *C. jejuni *81-176 increased the expression of epithelial IL-8 mRNA more than two-fold (Table 4). In contrast, IL-8 mRNA expression in monolayers treated with non-pathogenic *E. coli *HB101 (0.94 ± 0.17 fold) was similar to that of the sterile broth control (assigned a value of 1). IL-8 mRNA expression was higher in epithelial cells treated with isolates from AFLP cluster 1 compared to cells treated with AFLP cluster 2 isolates (5.03 ± 0.49 fold versus 3.80 ± 0.30 fold, respectively; *P *= 0.04). Mean IL-8 expression did not differ between *C. concisus *isolates belonging to genomospecies A and B (4.63 ± 0.57 fold versus 4.27 ± 0.35 fold, respectively; *P *= 0.62), nor between isolates from healthy and diarrheic humans (4.44 ± 0.72 fold versus 4.12 ± 0.29 fold, respectively; *P *= 0.64). Interleukin-8 expression was not correlated with invasion (R^2 ^= 0.002; *P *= 0.87) or translocation (R^2 ^= 0.14; *P *= 0.19).

**Table 4:** Expression of interleukin 8 mRNA in T84 monolayers inoculated with *Campylobacter concisus *isolates^a^.

Isolate	AFLP cluster	IL-8 mRNA expression (fold induction^b^)
CHRB2004	1	4.65 ± 1.82
CHRB3287	1	6.13 ± 1.14
CHRB2011	1	5.76 ± 1.16
CHRB3290	1	3.35 ± 0.63
CHRB1609	1	5.28 ± 1.77
		
CHRB1794	2	3.92 ± 0.91
CHRB6	2	4.53 ± 0.89
CHRB1569	2	4.11 ± 0.93
CHRB2691	2	3.49 ± 1.51
CHRB2370	2	5.46 ± 1.67
CHRB2050	2	2.61 ± 1.01
CHRB563	2	3.92 ± 2.51
CHRB3152	2	3.75 ± 0.42
CHRB3235	2	2.30 ± 0.25
		
LMG7788	1	4.53 ± 0.81
*C. jejuni *81-176	--	6.55 ± 1.35
*E. coli *HB101	--	0.94 ± 0.17

^a ^Data are means ± SEM, n = 3.

^b ^Expression of IL-8 relative to basal level of expression (assigned a value of 1). Data were normalized using the housekeeping gene C1orf33f.

### Toxin genes

The *CDT B *gene was not detected in any of the *C. concisus *isolates, but was present in *C. jejuni *81-176 (Additional file 3). The *zot *gene was detected in 80% of *C. concisus *isolates from healthy humans (i.e., four of five isolates), 22% of isolates from diarrheic humans (i.e., two of nine isolates), and the type strain. The *S-layer RTX *gene was present in *C. concisus *CHRB3287 and CHRB2004, although amplification was weak for the latter isolate. The *zot *and *S-layer RTX *genes were not detected in *C. jejuni *81-176.

## Discussion

The observed high level of genetic diversity amongst the isolates of *C. concisus *is in agreement with previous studies [1,2,10], and highlights the complex nature of this species. Cluster analysis of AFLP profiles indicated that the isolates examined in the current study comprised at least two distinct clusters. Similarly, Aabenhus et al. [1] denoted four AFLP clusters among 62 *C. concisus *isolates of which the majority (n = 56) were assigned to one of two main clusters. Results of PCR assays targeting the 23S rRNA and *cpn*60 genes largely corresponded with the AFLP grouping, and lend support to the suggested genetic relationship between the isolates.

As *C. concisus *is a common inhabitant of the oral cavity, it is to be expected that it may be isolated from both healthy and diarrheic individuals. Examining isolates from healthy individuals, it was observed that the majority of isolates belonged to genomospecies A and their AFLP profiles clustered together (AFLP cluster 1) along with the type stain of oral origin. This AFLP cluster also included one genomospecies A isolate (CHRB 1609) from a diarrheic individual. Further studies are needed to determine whether this group of isolates represents inhabitants of the oral cavity that have survived gastro-intestinal transit or whether they are intestinal-associated.

The majority (94%) of isolates from diarrheic individuals were assigned to ALFP cluster 2. Among these isolates, 71% were assigned to genomospecies B, while only 11% of diarrheic isolates belonged to genomospecies A. Engberg et al. [2] reported a similar predominance of genomospecies B isolates among diarrheic fecal isolates, of which 33% and 67% were assigned to genomospecies A and B, respectively. Likewise, Aabenhus et al. [1] reported that 34% and 53% of fecal isolates from diarrheic patients were assigned to genomospecies A and B, respectively. Our observation that isolates from genomospecies B were exclusively obtained from diarrheic individuals suggests a potential role for these isolates in intestinal disease. A comparative molecular examination of strains belonging to genomospecies A and B may shed light on their respective pathogenic potential.

Examination of the pathogenic properties amongst *C. concisus *isolates determined that epithelial invasion and translocation were higher for isolates assigned to AFLP cluster 2 (of which 94% were from diarrheic individuals). Additionally, epithelial translocation was higher for isolates belonging to genomospecies B (of which all isolates were from diarrheic individuals). This is of potential clinical relevance as invasiveness and translocation ability are the only factors definitively correlated with enteritis in *C. jejuni*-infected patients [26] and are likely associated with inflammatory responses and occasional bacteraemia observed with *C. concisus *infections [27]. To our knowledge, this is the first study to report differences in pathogenicity between the two main *C. concisus *genomospecies, further supporting the likelihood that isolates belonging to AFLP cluster 2/genomospecies B incite enteritis in humans.

All of the clinical *C. concisus *isolates examined in the current study caused hemolysis of sheep erythrocytes, consistent with previous observations of hemolytic phospholipase activity in all *C. concisus *genomospecies A and B isolates from diarrheic children [20]. As such, hemolytic activity appears to be a general characteristic of this species. Hemolysins are involved in pathogenesis and host colonization in other taxa [28], thus it was an unexpected observation that *C. concisus *genomospecies A isolates exhibited greater mean hemolysis than isolates belonging to genomospecies B. We also observed that hemolytic activity by *C. concisus *was inversely correlated with epithelial adherence and invasion. *Staphylococcus aureus *exhibits a similar inverse correlation that is attributed to interference of its α-hemolysin with epithelial β1-integrins that mediate host-cell interactions [29]. Moreover, lower amounts of α-hemolysin are produced by invasive *S. aureus *isolates from endocarditis patients compared to less-invasive isolates from open wounds [30]. Whether *C. concisus *hemolysin also interferes with epithelial receptors that promote adherence and invasion is unknown, and additional studies are warranted.

Another unexpected finding was that isolates from healthy individuals induced greater mean epithelial DNA fragmentation and metabolic activity compared to those from diarrheic individuals, and these variables were positively correlated. DNA fragmentation is used as an indicator of cell death. The two primary modes of cell death, namely apoptosis and necrosis, can be distinguished on the basis of physiologic features. DNA fragmentation can be present in both processes; however, during apoptosis, cell membranes typically remain intact, whereas during necrosis, cellular integrity is rapidly disrupted leading to the release of cytoplasmic contents (including lactate dehydrogenase) into the surrounding environment ("cytotoxicity"). Based on this definition, four isolates from healthy individuals (CHRB2004, CHRB3235, CHRB3287, and CHRB3290) and two isolates from diarrheic humans (CHRB2370 and CHRB3152) induced cell death consistent with apoptosis. While apoptosis can be mediated via CDT [18], it is unlikely that CDT was involved in the apoptosis given that we did not detect the CDTB gene in any of the strains included in our study. Furthermore, we did not find any genes with similar sequence to the CDTB gene using a BLAST search of the published *C. concisus *genome (NCBI accession number NC_009802), indicating that other factors (i.e. opposed to the CDT) may be responsible. The role that *Campylobacter*-induced epithelial cell death plays in pathogenesis is currently poorly understood; hence, the clinical significance of these findings for *C. concisus *remains to be determined.

Metabolic activity can be measured using the MTT assay in which metabolically active epithelial cells reduce a yellow tetrazolium salt (MTT) to purple formazan crystals that can be spectrophotometrically quantified. All of the isolates that we examined, except one isolate that caused epithelial sloughing (CHRB6), induced higher MTT values (> 130%) than the control, indicating that epithelial metabolic activity is increased by *C. concisus*. Some clinical strains of *C. jejuni *have also been reported to cause similar increases in epithelial MTT values [31]. Given the short incubation period for the MTT assay, we conclude that the increased values most likely reflect an increase in metabolic activity due to cellular stress rather than an increase in epithelial cell numbers due to proliferation. The observed correlation between metabolic activity and DNA fragmentation may be a consequence of the increased energy demands required to sustain the apoptotic process (i.e., apoptotic DNA fragmentation is an ATP-dependent process [32]).

The chemokine, IL-8 is a major mediator of inflammation. In the current study, all *C. concisus *isolates induced transcription of IL-8 in epithelial monolayers (> 2-fold) as has been previously reported for *C. jejuni *[19] and *C. concisus *[33]. *Campylobacter jejuni *induces epithelial IL-8 secretion by at least two independent mechanisms, one of which requires invasion and the other that is CDT-dependent [19,34]. We observed that induction of IL-8 transcription by *C. concisus *was not correlated with invasion. Man et al. also recently showed that three *C. concisus *strains stimulated production of IL-8 in intestinal epithelial irrespective of their invasive ability [33]. Thus in contrast to *C. jejuni*, it appears that factors other than invasion or CDT (which appears to be lacking in this species) are responsible for the up-regulation of IL-8 incited by *C. concisus*. The observation that expression of IL-8 mRNA was greater in epithelial cells treated with isolates from AFLP cluster 1 compared to isolates from cluster 2 was unexpected and suggests that these isolates may have pathogenic potential.

We identified genes encoding S-layer RTX and the zonnula occludins toxin in some of the isolates, confirming initial reports of these toxin genes in *C. concisus *[21]. Surprisingly, the *zot *gene was more prevalent in isolates from healthy (80%) compared to diarrheic (22%) humans. The clinical significance of this observation remains to be determined. Although Zot has been shown to disrupt epithelial tight junctions, we did not observe any changes in permeability or TER of epithelial monolayers throughout the 3 h incubation period for any of the isolates. This is contrary to the observation of Man et al., that *C. concisus *caused increased epithelial permeability, decreased TER, and loss of membrane-associated zonnula occludens and occludin in epithelial monolayers [33]. Possible reasons for this discrepancy include variation in methodology between the two studies (i.e., Man et al. inoculated Caco-2 cells with an MOI of 200, and assessed barrier function 6 h-post inoculation.).

## Conclusion

In summary, two main genomospecies were identified among fecal isolates of *C. concisus *from healthy and diarrheic individuals. The genomospecies differed with respect to clinical presentation and pathogenic properties, which is consistent with the hypothesis that certain genomospecies have different pathogenic potential. AFLP cluster 2 was predominated by isolates belonging to genomospecies B and those from diarrheic individuals. Isolates from this cluster displayed higher mean epithelial invasion and translocation than cluster 1 isolates, consistent with a potential role in inflammatory diarrhea and occasional bacteraemia. In contrast, isolates assigned to AFLP cluster 1 belonged to genomospecies A and were predominantly (but not strictly) isolated from healthy individuals. Isolates assigned to this cluster induced greater expression of epithelial IL-8 mRNA and more frequently contained genes coding for the zonnula occludins toxin and the S-layer RTX. Furthermore, isolates from healthy individuals induced greater apoptotic DNA fragmentation and increased metabolic activity than did isolates from diarrheic individuals, and isolates assigned to genomospecies A (of which the majority were from healthy individuals) exhibited higher haemolytic activity compared to genomospecies B isolates. This suggests that isolates from this cluster may also cause disease, albeit via different mechanisms than isolates from AFLP cluster 2. AFLP cluster 1 contains a reference strain isolated from the oral cavity, thus it is possible that this cluster contains isolates that are primarily periodontal pathogens.

While *in vitro *pathogenicity assessments are informative, they do not necessarily correspond with the ability of an isolate to cause disease *in vivo*. Clearly, further studies, particularly *in vivo*, are needed to confirm that these genetically distinct groups of *C. concisus *indeed differ in their ability to cause intestinal disease. In this regard, comparative genomic and pathogenicity examinations using animal models have been initiated.

## Methods

### Bacterial isolates and growth conditions

A total of 23 *C. concisus *isolates recovered from different individuals were used in this study (Table 1). These included five isolates recovered from the stools of healthy volunteers (i.e., individuals that exhibited no recent symptoms of enteritis), 17 isolates obtained from stools of diarrheic humans, and the type strain of *C. concisus *isolated from the oral cavity of a healthy human (LMG7788; = CCUG 13144; = ATCC 33237). Isolates were collected from people residing in the Chinook Health Region of Southwestern Alberta, Canada. These isolates were originally collected as part of a larger study [35]. Scientific and ethics approval for stool collection was obtained from the Regional Ethics Committee of the former CHR and from the University of Lethbridge Human Subject Research Committee. *Campylobacter jejuni *81-167 [36] was used as a positive pathogen control for all pathogenicity assays. In addition, the non-pathogenic *Escherichia coli *HB101 was used as a negative pathogen control for measuring epithelial IL-8 expression in response to the presence of bacteria. Isolates were stored at -80°C in Columbia broth (Difco, Detroit, MI) containing 40% glycerol. With the excepiton of *E. coli *which was grown in an aerobic enviornment, inocula of *C. concisus *for cell culture assays were prepared by growing isolates for 14-16 h in Columbia broth (37°C, 100 rpm) in a microaerobic atmosphere (consisting of 5% O_2_, 10% CO_2_, 30% H_2 _and balance nitrogen).

### 16S rRNA gene sequence

Genomic DNA was extracted using a DNAeasy Tissue kit (Qiagen Inc., Mississauga, ON) according to the manufacture's instructions. The 16S rRNA gene was PCR amplified using the primers UNI27F and UNI1492R [37] (Table 5) and the resultant product was used as template for sequencing. A BigDye Terminator kit (Applied Biosystems, Foster City, CA) along with universal primers (Table 5) were used for sequencing the near full-length 16s rRNA gene according to the manufacturer's instructions. Sequence reactions were separated with an ABI 3130 automated DNA sequencer (Applied Biosystems). Sequences were analyzed using Sequencher software (Gene Codes, Ann Arbor, MI) and compared directly with the NCBI non-redundant nucleotide database using BLASTN.

**Table 5:** Primers and adaptors used in this study.

Target^a^	Primer/Adaptor	Sequence (5' to 3')	Size (bp)	Reference
--	Bgl II adaptor1	CGGACTAGAGTACACTGTC	--	[38]
--	Bgl II adaptor2	GATCGACAGTGTACTCTAGTC	--	[38]
--	Csp6 I adaptor1	AATTCCAAGAGCTCTCCAGTAC	--	[38]
--	Csp6 I adaptor2	TAGTACTGGAGAGCTCTTGG	--	[38]
--	BLG2F-0	6-fam-GAGTACACTGTCGATCT	--	[38]
--	CSP61-A	GAGCTCTCCAGTACTACA	--	[38]
				
Universal 16S rRNA gene	UNI27F	AGAGTTTGATCCTGGCTCAG	--	[37]
	UNI338F	ACTCCTACGGGAGGCAG	--	[37]
	UNI1100R	AGGGTTGCGCTCGTTG	--	[37]
	UNI1492R	TACGG(C/T)TACCTTGTTACGACT	--	[37]
				
*C. concisus *23S rRNA gene	MUC1 (forward)	ATGAGTAGCGATAATTGGG	--	[11]
	CON1 (reverse)	CAGTATCGGCAATTCGCT	306	[11]
	CON2 (reverse)	GACAGTATCAAGGATTTACG	308	[11]
				
*C. concisus cpn *gene (primary primers)	Ccon-cpn_66f	TATCGAAGTGAAACGTGGCA	357	[35]
	Ccon_cpn_423r	GCTCAAGCACTGGCAATAAG	--	[35]
				
*C. concisus cpn *gene (nested primers)	Ccon_cpn_72f	AGTGAAACGTGGCATGGATA	270	[35]
	Ccon_cpn_342r	GCATCTTTTCAGGGTTTGTG	--	[35]
				
*C. concisus S-layer RTX *gene (YP_001465940.1)	FCCC13826_1838	ACAGGCCATAAGTGGATTGC	374	This study
	RCCC13826_1838	CCGTCATAGTGGGCTCTCAT	--	This study
				
*C. concisus zot *gene (YP_001467422)	FCCC13826_2075	TGCAAACCCTTTGTGATGAA	355	This study
	RCCC13826_2075	CATGAGCCAGCTCAATCAAC	--	This study
				
Human *interleukin 8 *gene (NM_000584)	hIL-8f	TTTTGCCAAGGAGTGCTAAAGA	194	PB ^b^
	hIL-8r	AACCCTCTGCACCCAGTTTTC	--	PB ^b^
				
Human *C1orf33 *gene (NM_016183)	hC1orf33f	TCCAAGCGCGACAAGAAAGT	102	PB ^b^
	hC1orf33r	GTAGGTGTCCACACATTTCCG	--	PB ^b^
				
*C. jejuni CDT B *gene (U51121)	P5	GAATCCGTTGGCACTTGGAATTTGCAAGGC	495	[40]
	P6	GGATTCGTTAAAATCCCCTGCTATCATCCA	--	[40]

^a ^GenBank or NCBI protein accession number indicated in brackets.

^b ^Primers sequences were obtained from the PrimerBank database http://pga.mgh.harvard.edu/primerbank/index.html

### Amplified fragment length polymorphism analysis

*Campylobacter concisus *isolates were genotyped using the AFLP protocol described by Kokotovic and On [38]. Briefly, genomic DNA (125 ng) was digested with Cps6I (10 U) in Y+/Tango Buffer (MBI) for 1 h at 37°C. BglII (10 U) was then added, and digestion was continued for one additional hour. Restriction site-specific adaptors (Table 5) were then ligated to the digested fragments for 2 h at room temperature. PCR amplification of the ligation mixture (diluted 10-fold) was carried out using primers BGL2F-0 and CSP6I-A (Table 5) for 35 cycles with an annealing temperature of 54°C. The final products were separated with an ABI 3130 automated DNA sequencer (Applied Biosystems). To analyze AFLP profiles, fragments ranging from 75 to 500 bp and the 500LIZ Genescan molecular mass standard were imported and compared using the BioNumerics 4.01 software (Applied Maths, Kortrijk, Belgium). Relationship of AFLP profiles ("curves") were inferred by use of the Pearson-product-moment correlation coefficient (applying 2% optimization) and clustered by the unweighted pair group with mathematical average (UPGMA) method. To ensure reproducibility, AFLP analysis was conducted twice for isolate, and one representative of each AFLP profile was used for cluster analysis.

### PCR for 23S rRNA, *cpn*60, *CDT B*, *S-layer RTX*, and *zot *genes

Primers for PCR are listed in Table 5. PCR amplification of the 23S rRNA gene was conducted according to the method of Bastyns et al. [11], except that the two reverse primers (CON1 and CON2) were used independently rather than as a mixture. Isolates amplifying with either MUC1/CON1 or MUC1/CON2 primers were assigned to genomospecies A or B, respectively. *Campylobacter concisus*-specific nested-PCR amplification of the chaperonin gene (*cpn*60) was conducted using the primers Ccon-cpn_66f and Ccon_cpn_423r for 25 cycles with an annealing temperature of 53°C [35]. The resultant PCR product was used as a template for a second round of PCR with the nested primers Ccon_cpn_72f and Ccon_cpn_342r for 30 cycles with an annealing temperature of 53°C. PCR for the *CDT B *gene was conducted using the primers P5 and P6 (Table 5) for 30 cycles with an annealing temperature of 65°C. PCR for the *S-layer RTX *gene was conducted using the primers FCCC13826_1838 and RFCCC13826_1838 (Table 5) for 30 cycles with an annealing temperature of 58°C. PCR for the *zot *gene was conducted using the primers FCCC13826_2075 and RFCCC13826_2075 for 30 cycles with an annealing temperature of 56°C.

### Intestinal epithelial cell culture and inoculation

T84 human colonic epithelial cells (passages 7 to 20; ATCC, Manassas, VA) were grown in DMEM/Ham F-12 plus 10% fetal bovine serum, 200 mM L-glutamine, 100 U/ml penicillin, 100 μg/ml streptomycin, 80 μg/ml tylosin (all from Sigma, Oakville, ON), and incubated at 37°C and 5% CO_2_. For cell culture assays, confluent T84 monolayers were washed twice and media was replaced with antibiotic-free DMEM/Ham F12. Monolayers were inoculated with sterile Columbia broth (= control) or *Campylobacter *to achieve a multiplicity of infection of 100 CFU per epithelial cell, and incubated for 3 h at 37°C. Due to the intensive nature of the assays for assessment of pathogenic potential (i.e., adherence, invasion, translocation, hemolytic ability, and cytotoxicity), representative isolates of *C. concisus *from diarrheic and healthy humans were examined for pathogenicity (n = 5 from AFLP cluster 1, n = 9 from AFLP cluster 2).

### Adherence and invasion

T84 enterocyte monolayers were grown in 24-well plates and inoculated as described above. Following incubation, monolayers were washed three times with PBS. To assess adherence, monolayers were lysed with 0.1% Triton X-100 in PBS for 10 min at room temperature on an orbital shaker. Following lysis, bacteria were enumerated by plating ten-fold serial dilutions onto Karmali agar (Oxoid, Nepean, ON). Invasion was determined using a gentamicin protection assay. After incubation, monolayers were washed three times with PBS. Monolayers were then incubated for 2 h with fresh tissue culture medium containing gentamicin (500 μg/ml) to kill extracellular bacteria as previously described [39]. Following incubation, monolayers were washed, lysed and bacteria were enumerated as for the adherence assay. A preliminary experiment was conducted to ensure that a bactericidal concentration of gentamicin was used for the invasion assay.

### Translocation and epithelial permeability

T84 cells were seeded onto Transwell filters at 4 × 10^5 ^cells/filter (5 μm pore size, 1.13 cm^2^; Costar, Corning Inc. Corning, NY) and cultured as described above. Transepithelial electrical resistance (TER) was monitored with an electrovoltohmeter (World Precision Instruments, Sarasota, FL), and monolayers were used at confluence (TER >1000 Ω × cm^2^). Monolayers were inoculated as described above. Following incubation, the basolateral medium was serially diluted, spread onto Karmali agar and incubated microaerobically at 37°C. Permeability was assayed as described previously [25]. Briefly, monolayers were washed with Ringer's solution, and a 3 kDa FITC-dextran probe (500 μl, 100 mM in Ringer's solution; Molecular Probes, Eugene, OR) was added to the apical compartment and 1 ml of Ringer's solution was also added to the basal compartment. Following incubation for 3 h at 37°C, samples were collected from the basal compartment and absorbance at 485 nm was measured.

### Hemolysis

Hemolysis of sheep erythrocytes was measured as previously described [20]. In brief, *C. concisus *cells cultured in Columbia broth as described above were centrifuged (8000 × g, 3 min) and cell pellets were washed with sterile PBS, suspended in PBS to 1 × 10^9 ^CFU/ml, and then serially diluted 2-fold in PBS. Equal volumes (100 μl) of cell suspension and sheep erythrocytes (2% vol/vol in PBS) were mixed in a U-bottom 96-well plate. The plate was then incubated at 37°C under microaerobic conditions for 18 h. A comparative negative control (without bacteria) was also incubated under similar conditions. A positive control for total hemolysis (100%) was performed by replacing the same volume of bacterial cell suspension with distilled water. After incubation, the tubes were centrifuged at 1000 × g for 5 min, and the OD_490 _of the supernatants for the 1/3 dilution were measured. Data were reported as the percent total hemolysis of sheep erythrocytes (compared to the positive control).

### DNA fragmentation, cytotoxicity, and metabolic activity

T84 monolayers were grown in 24-well plates and inoculated as described above. Control monolayers were also treated with camptothecin (4 μM), hydrogen peroxide (H_2_O_2_, 0.5 mM), or sterile broth. Following incubation, DNA fragmentation was measured using a Cellular DNA Fragmentation ELISA kit (Roche Applied Science, Laval, QC) according to the manufacturer's protocol. Lactate dehydrogenase released into the surrounding tissue culture was measured using a Cytotoxicity Detection kit (Roche) according to the manufacturer's protocol. Metabolic activity (i.e. MTT assay) was measured using a Cell Proliferation Kit I (Roche) according to the manufacturer's protocol, except that gentamicin (500 μg/ml) was incorporated into the MTT solution.

### Interleukin-8 real-time quantitative PCR

T84 monolayers were grown in six-well plates and inoculated with *C. concisus *and *C. jejuni *as described above. In addition, monolayers were inoculated at an MOI of 100 with *E. coli *HB101. Following incubation, the culture medium was removed and replaced with RNAlater (3 ml/well; Qiagen), and cells were stored at 4°C until processed for RNA extraction (< 1 week). Total RNA was isolated using the RNeasy mini kit (Qiagen), according to the manufacturer's protocol. RNA was reverse transcribed using a QuantiTect reverse transcription kit (Qiagen) according to the manufacturer's protocol. PCR was conducted using an Mx3005P Stratagene thermocycler (Stratagene, Cedar Creek, TX). All PCR reactions were carried out in 20 μl volumes and contained 1X QuantiTect SYBR Green PCR Master Mix (Qiagen), forward and reversed primers (0.5 μM each; Table 5) and 2 μL of RT reaction. Amplification conditions were 1 cycle at 95°C for 15 min followed by 40 cycles of 94°C for 15 s, 60°C for 30 s, and 72°C for 30 s. Melting curve analysis was conducted over a range of 55 to 95°C to assess specificity of amplification. Interleukin-8 expression was normalized to the housekeeper gene, C1orf33, and fold changes in expression relative to the sterile-broth control was calculated using the 2^-ΔΔCT ^method.

### Statistical analysis

Experiments were conducted at least three times on separate occasions (i.e., replicates). Each assay was conducted at least in duplicate (i.e., observations), and the mean value was used for analysis. Data are expressed as mean ± SEM. All statistical calculations were performed with GraphPad InStat v.3.06 software (GraphPad Software Inc., San Diego, CA). Data with three or more treatments were compared by one way analysis of variance, followed by the protected Tukey-Kramer multiple comparison test. Data with two treatments were compared using an unpaired Student's *t*-test. Regression analysis was performed using Pearson correlation analysis. Statistical significance was established at *P *< 0.05.

## Authors' contributions

LDK participated in the design of the study, performed experiments, conducted data analysis, and drafted the manuscript. GDI participated in the design of the study and edited the manuscript. All authors approved the final manuscript.

## Supplementary Material

Additional file 1 **Dendrogram of *C. concisus *AFLP profiles demonstrating reproducibility between duplicate independently-prepared samples**. AFLP profiles were derived using the unweighted-pair group average linkage of Pearson-product-moment correlation coefficients from 22 *Campylobacter concisus *fecal isolates (designated CHRB) and the type strain (LMG7788). The bar indicates percentage similarity. *, isolates for which only a single profile was analyzed. Additional file 1 contains a figure. Click here for file

Additional file 2 **Transepithelial resistance (TER) and FITC-dextran permeability for confluent, polarized T84 monolayers inoculated with *Campylobacter concisus *isolates^a^**. Additional file 2 contains a table. Click here for file

Additional file 3 **PCR screening of genes coding for cytolethal distending toxin (CDT), zonula occludens toxin (Zot), and S-layer RTX for *Campylobacter concisus *isolates**. Additional file 3 contains a table. Click here for file
