# Supplementary figures and images for: Comparative genotypic and pathogenic examination of Campylobacter concisus isolates from diarrheic and non-diarrheic humans
Source: BMC Microbiol. 2011 Mar 15;11:53. doi: 10.1186/1471-2180-11-53 (PMC3068073; doi:10.1186/1471-2180-11-53)

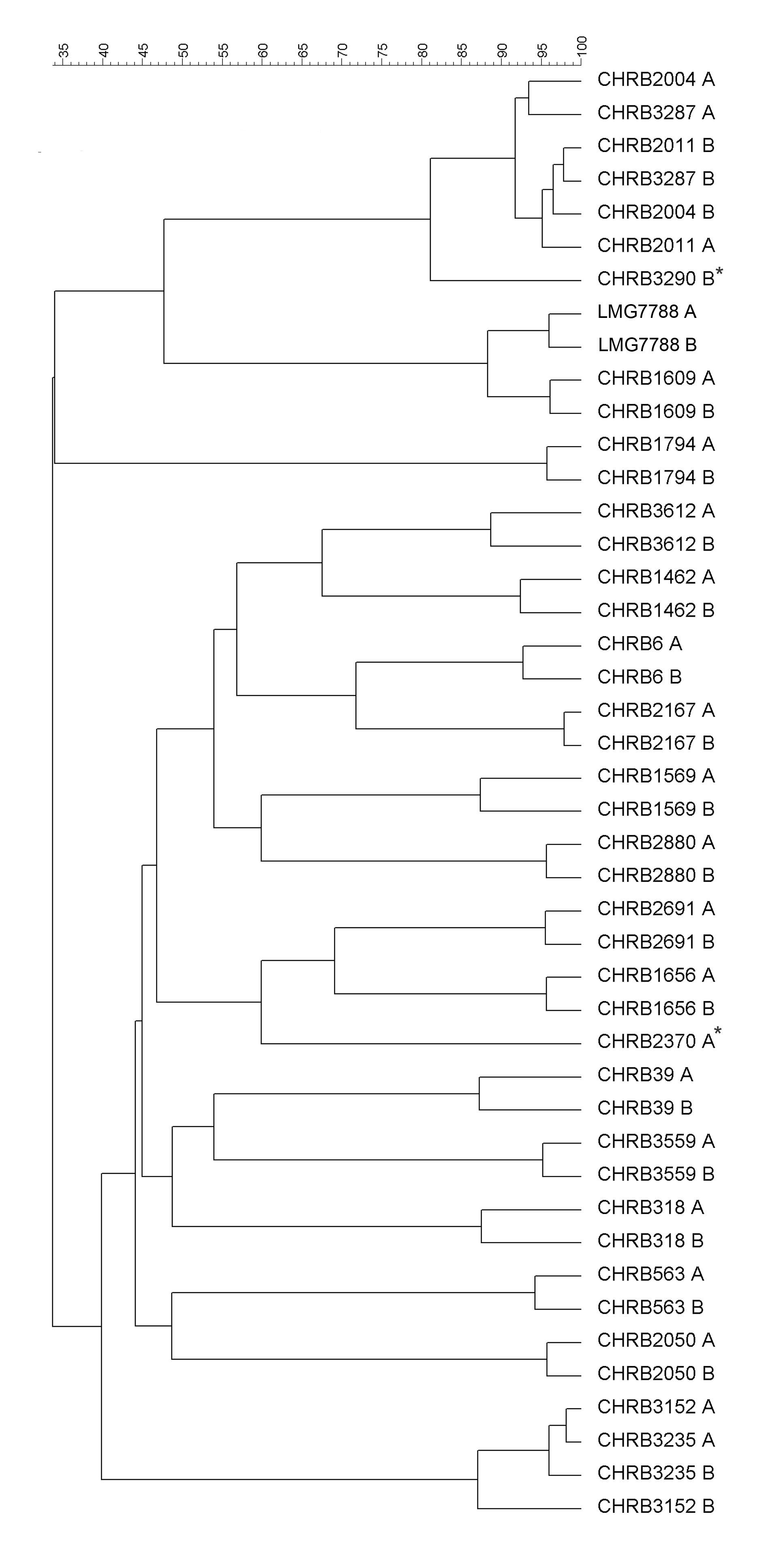

Supplement: Additional file 1 — Dendrogram of C. concisus AFLP profiles demonstrating reproducibility between duplicate independently-prepared samples. AFLP profiles were derived using the unweighted-pair group average linkage of Pearson-product-moment correlation coefficients from 22 Campylobacter concisus fecal isolates (designated CHRB) and the type strain (LMG7788). The bar indicates percentage similarity. *, isolates for which only a single profile was analyzed. Additional file 1 contains a figure. [file 1471-2180-11-53-S1.JPEG]
