# Supplementary material for: Comparative genotypic and pathogenic examination of Campylobacter concisus isolates from diarrheic and non-diarrheic humans
Source: BMC Microbiol. 2011 Mar 15;11:53. doi: 10.1186/1471-2180-11-53 (PMC3068073; doi:10.1186/1471-2180-11-53)
Supplement: Additional file 2 — Transepithelial resistance (TER) and FITC-dextran permeability for confluent, polarized T84 monolayers inoculated with Campylobacter concisus isolatesa. Additional file 2 contains a table. [file 1471-2180-11-53-S2.DOC]

**Additional file 2: Transepithelial resistance (TER) and FITC-dextran permeability for confluent, polarized T84 monolayers inoculated with *Campylobacter concisus* isolates^a^.**

**Isolate AFLP Initial TER Final TER FITC-dextran permeability**

**Cluster (Ω × cm^2^) (Ω × cm^2^) (% apical dextran/h)**

CHRB2004 1 1043 ± 3 1108 ± 18 0.25 ± 0.02

CHRB3287 1 1043 ± 0.7 1120 ± 10 0.24 ± 0.04

CHRB2011 1 1041 ± 4 1073 ± 4 0.22 ± 0.06

CHRB3290 1 1057 ± 10 1119 ± 33 0.22 ± 0.04

CHRB1609 1 1041 ± 11 1150 ± 29 0.21 ± 0.03

CHRB1794 2 1034 ± 11 1071 ± 1 0.22 ± 0.03

CHRB6 2 1050 ± 19 1107 ± 11 0.20 ± 0.02

CHRB1569 2 1033 ± 2 1111 ± 26 0.20 ± 0.04

CHRB2691 2 1024 ± 3 1133 ± 33 0.23 ± 0.01

CHRB2370 2 1048 ± 1 1075 ± 1 0.19 ± 0.01

CHRB2050 2 1038 ± 2 1076 ± 3 0.23 ± 0.03

CHRB563 2 1043 ± 3 1108 ± 18 0.25 ± 0.02

CHRB3152 2 1048 ± 3 1061 ± 9 0.21 ± 0.04

CHRB3235 2 1028 ± 2 1067 ± 3 0.23 ± 0.03

LMG7788 1 1065 ± 13 1096 ± 6 0.23 ± 0.03

*C. jejuni* 81-176 — 1043 ± 13 1077 ± 3 0.25 ± 0.02

Broth control — 1046 ± 15 1095 ± 20 0.24 ± 0.01

^a^ Data are means ± SEM, n = 3.
