# Supplementary material for: Comparative genotypic and pathogenic examination of Campylobacter concisus isolates from diarrheic and non-diarrheic humans
Source: BMC Microbiol. 2011 Mar 15;11:53. doi: 10.1186/1471-2180-11-53 (PMC3068073; doi:10.1186/1471-2180-11-53)
Supplement: Additional file 3 — PCR screening of genes coding for cytolethal distending toxin (CDT), zonula occludens toxin (Zot), and S-layer RTX for Campylobacter concisus isolates. Additional file 3 contains a table. [file 1471-2180-11-53-S3.DOC]

**Additional file 3: PCR screening of genes coding for cytolethal distending toxin (CDT), zonula occludens toxin (Zot), and S-layer RTX for *Campylobacter concisus* isolates.**

Isolate AFLP CDT Zot S-layer RTX

cluster

CHRB2004 1 – + w

CHRB3287 1 – + +

CHRB2011 1 – + –

CHRB3290 1 – + –

CHRB1609 1 – – –

CHRB1794 2 – + –

CHRB6 2 – + –

CHRB1569 2 – – –

CHRB2691 2 – – –

CHRB2370 2 – – –

CHRB2050 2 – – –

CHRB563 2 – – –

CHRB3152 2 – – –

CHRB3235 2 – – –

LMG7788 — – + –

*C. jejuni* 81-176 — + – –
